# Interfacial Binding Energy between Calcium-Silicate-Hydrates and Epoxy Resin: A Molecular Dynamics Study

**DOI:** 10.3390/polym13111683

**Published:** 2021-05-21

**Authors:** Xianfeng Wang, Wei Xie, Jun Ren, Jihua Zhu, Long-Yuan Li, Feng Xing

**Affiliations:** 1 Guangdong Provincial Key Laboratory of Durability for Marine Civil Engineering, College of Civil and Transportation Engineering, Shenzhen University, Shenzhen 518060, China; xfw@szu.edu.cn (X.W.); xw13671400570@163.com (W.X.); xingf@szu.edu.cn (F.X.); 2 Harbin Institute of Technology, School of Science, Shenzhen 518055, China; 3 School of Engineering, University of Plymouth, Plymouth PL4 8AA, UK; long-yuan.li@plymouth.ac.uk

**Keywords:** calcium-silicate-hydrates, epoxy resins, molecular dynamics, interfacial binding energy, self-healing concrete

## Abstract

Microcapsules encapsulated within epoxy as a curing agent have been successfully applied in self-healing materials, in which the healing performance significantly depends on the binding behaviour of the epoxy curing agent with the cement matrix. In this paper, the binding energy was investigated by molecular dynamics simulation, which could overcome the shortcomings of traditional microscopic experimental methods. In addition to the construction of different molecular models of epoxy, curing agents, and dilutants, seven models were established to investigate the effects of chain length, curing agent, and epoxy resin chain direction on the interfacial binding energy. The results showed that an increase of chain length exhibited had limited effect on the binding energy, while the curing agent and the direction of the epoxy significantly affected the interfacial binding energy. Among different factors, the curing agent tetrethylenepentamine exhibited the highest value of interfacial binding energy by an increment of 31.03 kcal/mol, indicating a better binding ability of the microcapsule core and the cement matrix. This study provides a microscopic insight into the interface behaviour between the microcapsule core and the cement matrix.

## 1. Introduction

As the most widely used building material worldwide [1], the safety and service life of concrete is important. Unfortunately, during its service life, microcracks or defects inevitably occur in concrete. Without proper control, this may lead to a serious impact on the structures, and further reduce the durability and shorten the service life of the structures [2,3]. To solve this issue, many scholars have attempted to repair microcracks by employing various methods [4,5,6,7]. Among them, according to the principle of bionics, self-healing concrete with microcapsules have been developed, which can be used to automatically locate and repair the microcracks inside concrete and further improve the durability of concrete structures [8]. Moreover, compared with traditional repairing technology, the microcapsule self-healing system exhibits the advantages of automatic repair, low cost, and renewability. In general, this self-healing system is designed based on three steps to achieve the repairing effect: (1) embedding the encapsulated healing agent in the cement matrix, (2) releasing the healing agent after the breaking of the container when the matrix produces microcracks, and (3) bonding the cementitious matrix with the cured healing agent to block the microcracks [9].

Currently, microcapsules with a urea-formaldehyde (UF) shell have been proven to be one of the most effective microcapsules for repairing concrete cracks [10,11]. According to research, the core of the microcapsules was prepared by a mixture of bisphenol A type epoxy resin (E-51), and diluent (*n*-butyl glycidyl ether, BGE) was used as the healing agent, while the MC120D or tetrethylenepentamine (TEPA) was mixed as an epoxy curing agent. When cracks inside the concrete occur, the microcapsules are broken, releasing the epoxy resin. Due to the capillary siphon effect caused by the cracks, the epoxy resin can quickly penetrate into the cracks and solidify with a curing agent to fill the cracks, so as to achieve the purpose of repairing the concrete cracks [9].

There are many factors affecting the repairing efficiency of microencapsulated self-healing concrete, among which, the bond strength between the healing agent and the cement matrix has been identified as one of the most important factors influencing the repairing efficiency of microcapsules. It is generally agreed that the interfacial bond strength is affected by curing agent type, dosage, curing temperature, and other factors. For example, Zhang et al. [12] investigated the factors influencing the bond strength between the microcapsule healing agent and the cement matrix, and found that the curing of the microcapsule core (epoxy resin) showed great influence on the healing effect, and the combination of two curing agents (MC120D and TEPA) offered better repairing performance than that of a single agent. Although the experimental methods provided insights into the bond strength between the microcapsule healing agent and the surface of the cementitious matrix, the results still faced the challenge that the investigation was only conducted on the macroscale, where the underlying mechanism on a molecular level was still constrained. Therefore, molecular dynamics (MD) simulation technology, which has been applied in the characterisation of the microstructure and microscale properties of cement-based materials, could offer an opportunity to study the binding behaviour of epoxy resin and cementitious materials [13,14].

MD is a method to obtain the phase trajectory of a molecular system by numerically solving the equations of motion and to count the structural characteristics and properties of the aiming system. In the study of cement and concrete, MD has been extensively applied to simulate the mechanical behaviour of cement materials at the molecular scale, particularly with calcium silicate hydrate (C–S–H), one of the most important hydration products of cement. Wang et al. [15] studied the interfacial shear strength between C–S–H and polymer fibres via the determination of shear strength, static molecular structure, and the dynamic characteristics of the interface, and they reported that the Ca atoms that existed at the interface played an important role on the interface bond cooperation by forming the O_CSH_–Ca–O_polymer_ connection. Hou et al. [16] applied MD to study the interface structure, kinetics, thermodynamics, and mechanical properties between C–S–H and a polymer. In the study, the polymer/C–S–H composite model with polyethylene glycol, polyvinyl alcohol, and polyacrylic acid, which were inserted into the nanochannel of C–S–H sheets, was constructed, and the calcium ions near the C–S–H surface were reported as the bridging substance between the polymer functional group and the oxygen in the silicate chain by forming O_S_–Ca–O_p_. Du et al. [17] studied the epoxy resin/cement interface by using a combined experimental analysis and molecular simulation with a C–S–H/resin interface model and measured the adhesion energy of the C–S–H/resin interface. The results showed that the polarity of the resin enhanced the attraction between positive ions (such as Ca^2+^) and water molecules to the resin molecules. It was reported that the interface failure was caused by brittle failure due to the simultaneous separation of epoxy atoms from the matrix, and the tensile strength of the interface was almost unaffected by unloading and reloading before reaching the failure strength. In summary, MD provides a powerful tool for studying the properties of cement materials at the molecular level, which can be used to explain the various properties of the cement paste, mainly the microstructure and mechanical properties, as well as the interface behaviour between the composite models [18,19,20,21]. However, in a microcapsule-based self-healing system, the factors that influence the interfacial properties of C–S–H/epoxy resins have not been investigated at the molecular scale, which requires the involvement of MD simulation. As stated previously, since the binding behaviour of the interface at two phases is important for indicating the repairing efficiency of the curing agent [12], it is essential to conduct the investigation on the binding behaviour between the organic curing agent and the inorganic cementitious matrix in a microcapsule-based concrete with the assistance of MD.

This paper aims to investigate the binding behaviour between tobermorite, the hydration product of cement and epoxy resin, from the perspective of the molecular level. To obtain a comprehensive understanding, the effects of epoxy chain length, curing agent type, and epoxy direction on the interfacial behaviour were investigated by determining the mean square displacement (MSD), the radial distribution function (RDF), and the interfacial binding energy of the established composite models between tobermorite and epoxy resin with different chemical structures.

## 2. Computational Methodology

### 2.1. Forcefield

The force field illustrates the interaction of an atom with its surrounding atoms, which is the core of mechanical simulation. In this study, the COMPASS force field is used to describe the interaction between atoms, which is an ab initio force field, and its algorithm is largely derived from the early force field, namely the CFF force field. The potential function of the COMPASS force field can be expressed in Equation (1) [22]:

(1) $$\begin{array}{l} E_{total}=\underset{b}{\sum }[k_{2}{\left(b-b_{o}\right)}^{2}+k_{3}{\left(b-b_{o}\right)}^{3}+k_{4}\left(b-b_{o}{)}^{4}\right]+\underset{\theta }{\sum }[k_{2}{\left(\theta -\theta_{0}\right)}^{2}\\ \;\;\;\;\;\;\;+k_{3}{\left(\theta -\theta_{0}\right)}^{3}+k_{4}{\left(\theta -\theta_{0}\right)}^{4}+\underset{\phi }{\sum }[k_{1}(1-cos\phi )\\ \;\;\;\;\;\;\;+k_{2}\left(1-cos2\phi \right)+k_{3}\left(1-cos3\phi \right)]+\underset{\chi }{\sum }k_{2}\chi^{2}+\underset{b,b^{\prime }}{\sum }k(b\\ \;\;\;\;\;\;\;-b_{o})\left(b^{\prime }-{b^{\prime }}_{0}\right)+\underset{b,\theta }{\sum }k\left(b-b_{o}\right)(\theta -\theta_{0})\\ \;\;\;\;\;\;\;+\underset{b,\phi }{\sum }\left(b-b_{0}\right)[k_{1}cos\phi +k_{2}cos2\phi +k_{3}cos3\phi ]\\ \;\;\;\;\;\;\;+\underset{\theta ,\phi }{\sum }\left(\theta -\theta_{0}\right)[k_{1}cos\phi +k_{2}cos2\phi +k_{3}cos3\phi ]\\ \;\;\;\;\;\;\;+\underset{b,\theta }{\sum }k\left(\theta^{\prime }-\theta_{0}^{\prime }\right)\left(\theta -\theta_{0}\right)+\underset{\theta ,\theta ,\phi }{\sum }k\left(\theta -\theta_{0}\right)\left(\theta^{\prime }-\theta_{0}^{\prime }\right)cos\phi \\ \;\;\;\;\;\;\;+\underset{i,j}{\sum }\frac{q_{i}q_{j}}{r_{ij}}+\underset{i,j}{\sum }\epsilon_{ij}[2(\frac{r_{ij}^{0}}{r_{ij}}{)}^{9}-3(\frac{r_{ij}^{0}}{r_{ij}}{)}^{6}] \end{array}$$

where b is the bond, θ is the angle, ϕ is the torsion angle, χ is the out-of-plane angle, q is the charge, and r the distance between two atoms. In this formula, the first seven terms are the bonding effect of atoms, which are: bond length, bond angle, dihedral angle, bond expansion–bond expansion coupling, bond expansion–bond angle bending coupling, bond expansion–dihedral angle twisting coupling, bond angle bending–dihedral angle torsion coupling, and bond angle bending–bond angle bending coupling, the latter two are non-bonding interactions of atoms, van der Waals and Coulomb interactions, respectively.

The COMPASS force field covers a wide range of covalent molecules, including polymers; organic molecules and small inorganic molecules; and non-covalent bond models covering a range of ionic materials including metals, metal oxides, and metal halides. It has been proven that the COMPASS force field shows good compatibility with cementitious materials [23,24,25,26].

### 2.2. Model Construction

**Figure 1:**
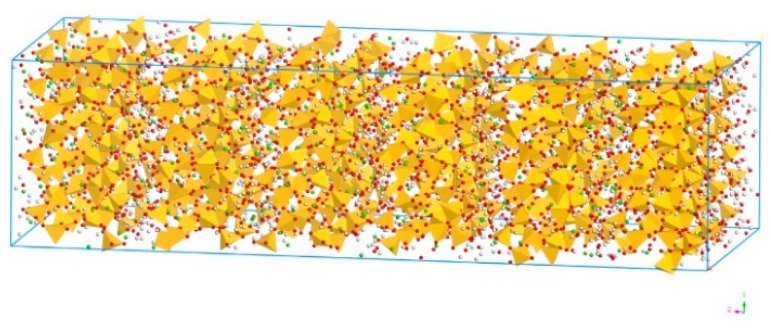
Molecular structure of tobermorite 14 Å (Color legend: hydrogen H (white); calcium Ca (green); oxygen O (red); silica Si (yellow polyhedral)).

The simulation in this paper was carried out using Biovia Materials Studio 2016 (Biovia Co., Shanghai, China). It has been widely agreed that tobermorite has a similar interlayer structure to C–S–H [27,28,29,30,31,32], therefore the model of tobermorite has been commonly applied to simulate C–S–H due to its complexity [33,34,35,36]. In this paper, based on the monoclinal ordered layer structure of the spatial group B11b and the chemical formula of Ca_5_Si_6_O_16_(OH)_2_•7H_2_O [37], the tobermorite 14 Å was modelled, which is shown in Figure 1. It should be noted that although the calcium atoms and water molecules randomly occupy the interlaminar space of tobermorite 14 Å, in this model, they were assumed to alternately reside in these positions in an ordered model, which is a common approach in the related study [37].

**Figure 2:**
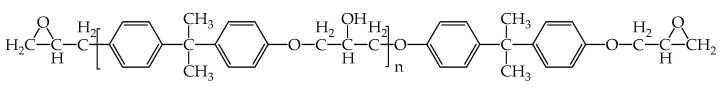
The chemical information of epoxy.

**Figure 3:**
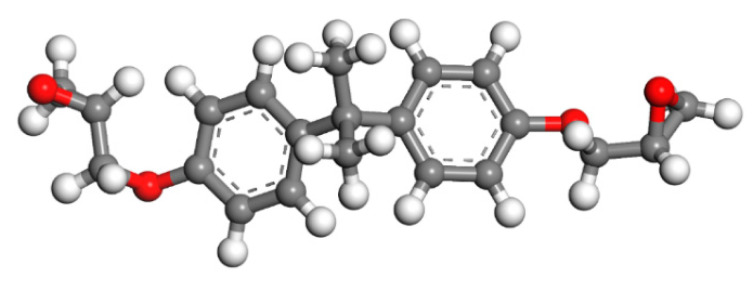
The model of E-51(*n* = 0; colour legend: hydrogen H (white); oxygen O (red); carbon C (grey)).

**Figure 4:**
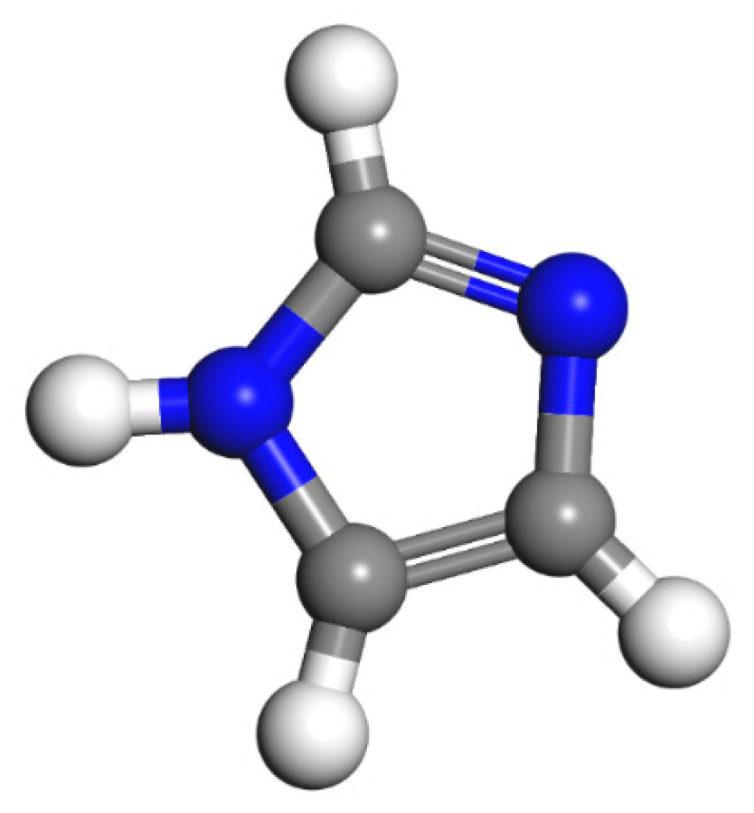
The model of MC120D (colour legend: hydrogen H (white); nitrogen N (blue); carbon C (grey)).

**Figure 5:**
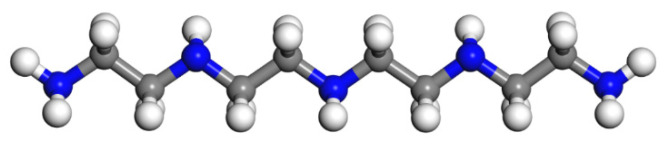
The model of TEPA (colour legend: hydrogen H (white); nitrogen N (blue); carbon C (grey)).

**Figure 6:**
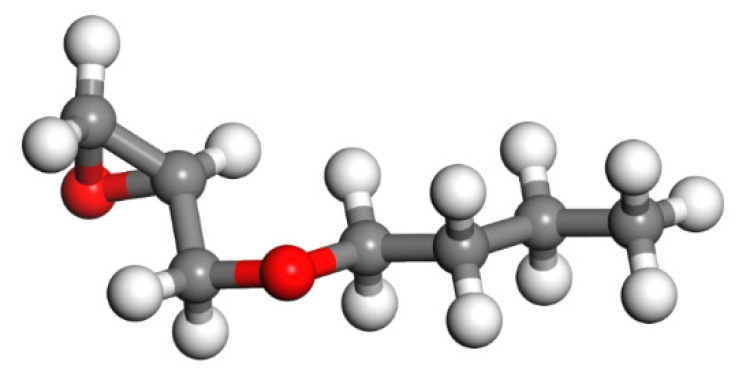
The model of BGE (colour legend: hydrogen H (white); oxygen O (red); carbon C (grey)).

**Figure 7:**
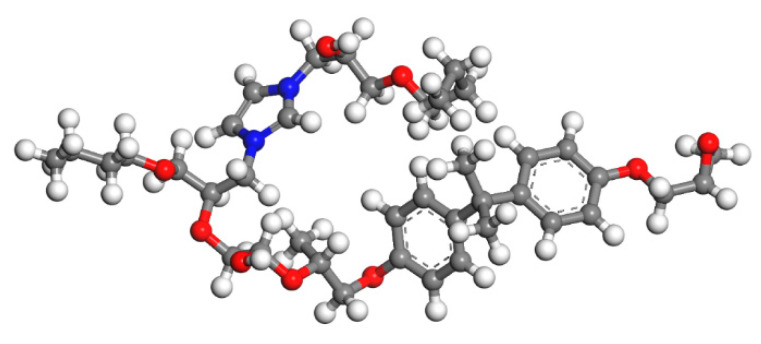
The model of epoxy resin cured with MC120D (colour legend: hydrogen H (white); oxygen O (red); carbon C (grey); nitrogen N (blue)).

**Figure 8:**
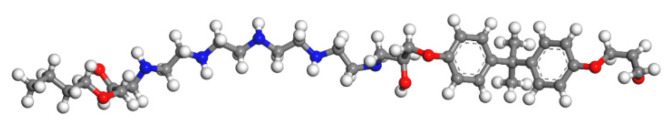
The model of epoxy resin cured with TEPA (colour legend: hydrogen H (white); oxygen O (red); carbon C (grey); nitrogen N (blue)).

Since epoxy is commonly used as a healing agent in self-healing systems [38,39], it was selected to simulate the binding behaviour with a cementitious matrix. As illustrated in Figure 2, the molecular chain of epoxy resin contains unique active groups and polar groups, such as epoxy groups, hydroxyl groups, and ether groups [40]. In view of the advantages of bisphenol A type epoxy resin such as high bond strength, wide bonding surface, good stability, low shrinkage, and good mechanical properties, E-51 was selected as the core of the microcapsule [41]. According to the literature, epoxies with *n* less than or equal to 2 were a fluid phase [12], therefore, *n* was set as 0, 1, 2 in the epoxy resin model. Moreover, in order to study the influence of the curing agent and chain length, six epoxy resin models were established, namely, the epoxy resin model without curing and the epoxy resin model after the reaction with diluent and curing agent. The model established according to the chemical structure formula of epoxy resin is shown in Figure 3. At room temperature, E-51 epoxy resin exhibited a high viscosity, which is unfavourable for the epoxy’s ability to flow into cracks after rupture, and requires a diluent to reduce the viscosity to infiltrate the crack surface. For a self-healing process, after the epoxy resin flows into the crack, a curing reaction then occurs with the curing agent, i.e., MC120D (Figure 4) and TEPA (Figure 5), while the diluent (BGE, as shown in Figure 6) may participate in the curing reaction. According to the curing reaction principle [42], the models of the cured epoxy resin with MC120D and TEPA were established, which are shown in Figure 7 and Figure 8, respectively.

### 2.3. Simulation

**Figure 9:**
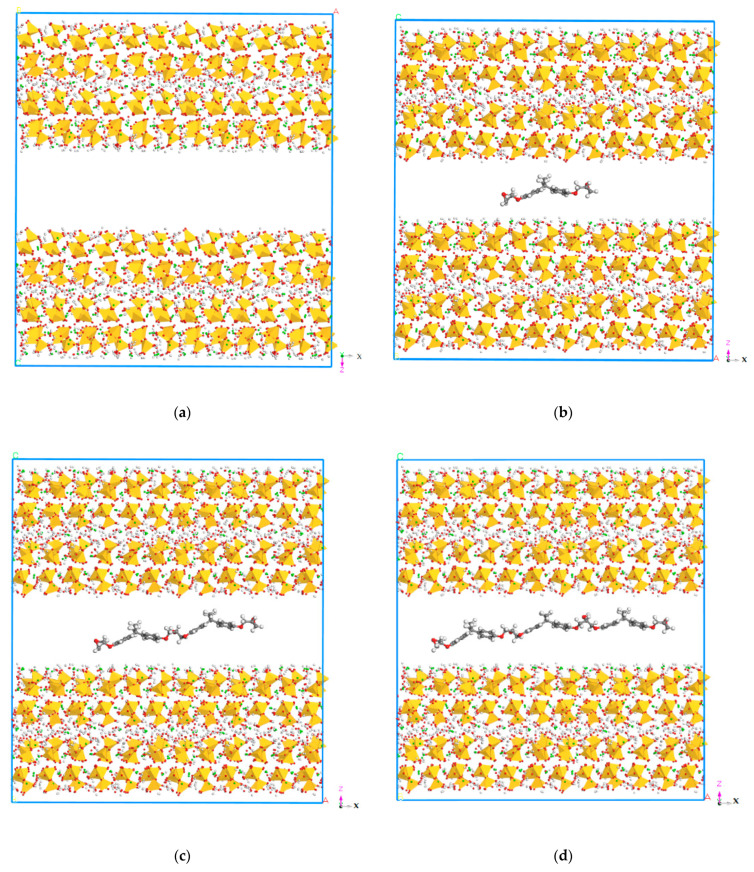

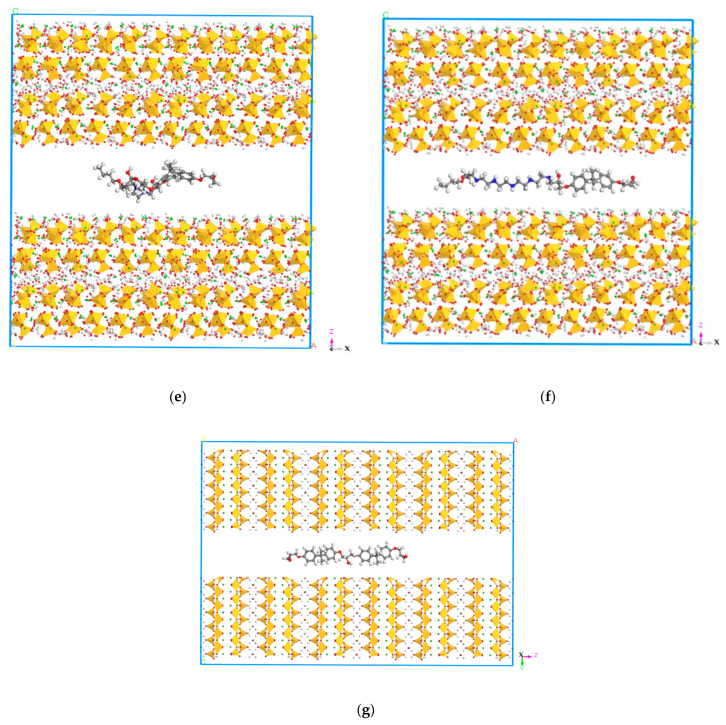
Molecular structures of the models. (**a**) Molecular structure of model 1. (**b**) Molecular structure of model 2. (**c**) Molecular structure of model 3. (**d**) Molecular structure of model 4. (**e**) Molecular structure of model 5. (**f**) Molecular structure of model. (**g**) Molecular structure of model 7.

In this study, a total of 7 models of composites were established, which are shown in Table 1. In order to optimize the tobermorite 14 Å structure, the smart algorithm was used for energy minimisation. To establish the interlayer structure, the cleave layer tool was used for the optimized tobermorite 14 Å along [0 0 1] (Z direction) and [0 1 0] (Y direction) to obtain the XY plane and XZ plane, respectively. Then, a three-dimensional space structure was created with vacuum space. In order to build the layered structure, the epoxy models were built as crystals with length c (along the Z direction) of 10 Å. The layered structure was built with layer 1 and layer 3 of tobermorite and layer 2 of epoxy placed in between. Therefore, the width of the crack in the final model was obtained as 14 Å after matching the model structure, which was in the range of 0.5 nm–100 nm as reported in C–S–H by Ma et al. [43]. As shown in Figure 9, the interlayer structure, in which tobermorite 14 Å was on both sides and epoxy resin was in the middle, was constructed. After the whole composite model was established, the structure of the whole model was optimised again.

During the entire MD simulation process, the time step was set as 1 fs and the temperature was fixed at 298 K. The temperature and pressure were monitored and controlled by Nose Thermostat and Berendsen Barostat. Meanwhile, periodic boundary conditions were used [44]. The entire simulation process was divided into two steps. First, the models were relaxed for 1000 ps in the *NPT* (*N*—number of atoms, *P*—pressure, *T*—temperature) isothermal–isobaric ensembles, in which the pressure was set to 0 GPa. Second, a further 2000 ps run was conducted in the *NVT* (*N*—number of atoms, *V*—volume, *T*—temperature) ensembles to output the result of MD simulation.

## 3. Results and Discussion

### 3.1. Structural Analysis

**Figure 10:**
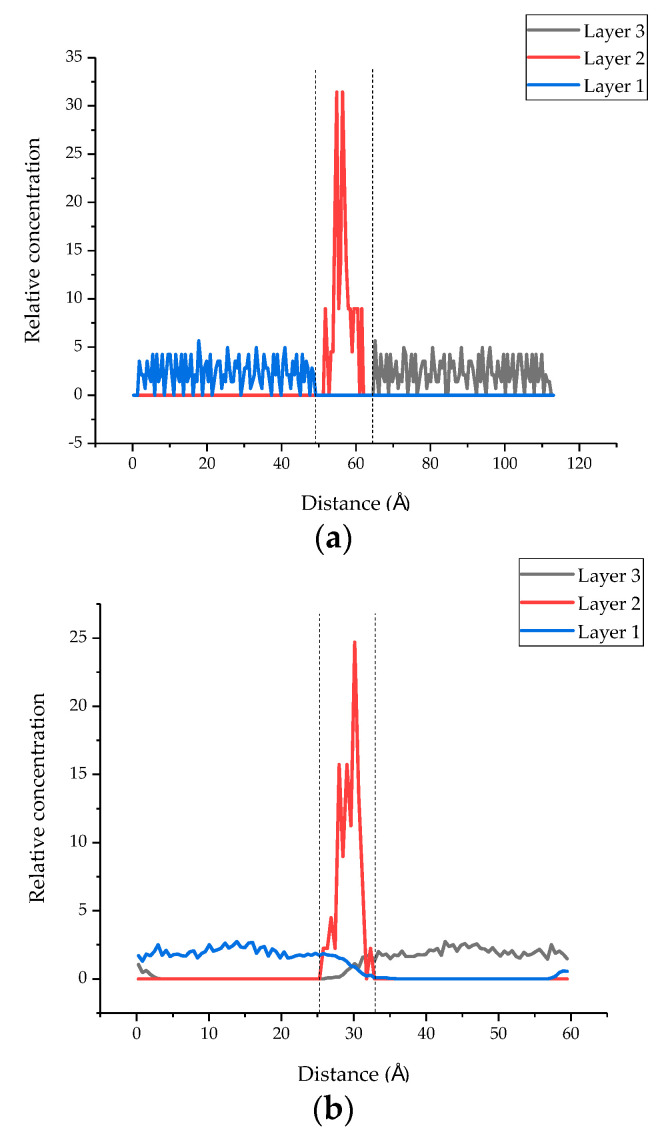
The relative concentration distribution before and after MD simulation (layer 1 and layer 3 are the tobermorite layers, and layer 2 is the epoxy resin layer). (**a**) The relative concentration distribution of the original model. (**b**) The relative concentration distribution after MD simulation.

The initial cell lengths in the Z direction before and after MD are listed in Table 2. It can be seen from the table that after MD simulation, the length of all models in the Z direction decreased, which could be due to the reduction in the layer spacing of each compound. Moreover, it should be noted that the cell length in the Z direction of the seven models all decreased after MD simulation, but the reduction in cell length may be attributed to the insertion of the epoxy resin chain, which was smaller than that of the model without epoxy resin. Moreover, as shown in Figure 10, the relative concentration distribution of the three layers of the overall model (the tobermorite layer on both sides and the epoxy resin layer in the middle, models 2 to 6) also reflects a similar phenomenon. In Figure 10a, the atoms of layer 2 were distributed between 51.74−61.52 Å, where there were no atoms of layer 1 and layer 3. On the contrary, the atoms of layer 2 were distributed between 25.80−32.32 Å and the atoms of layer 1 and layer 3 were distributed between 0−35.58 Å and 26.35−59.38 Å, from which we can see that the atoms of the three layers infiltrated each other after the MD simulation. These might be due to the molecular interaction between the different layers, resulting in the decrease in distance and the cracks between the models being reduced.

### 3.2. Mean Square Displacement

The binding energy mainly depends on the interaction between O and N in cured epoxy resin and Ca in tobermorite, which can be reflected by the mean square displacement (MSD) to indicate the repairing efficiency of the cured epoxy resin. The MSD is often used to analyse the displacement of atoms with time, and the estimation of the motion parameters can be obtained in Equation (2). MSD (*t*) could be applied to describe atoms deviating from their initial position as the function of time.

(2) $$<r^{2}\left(t\right)>=\frac{1}{N}\overset{N}{\underset{i=1}{\sum }}<|r_{i}\left(t\right)-r_{i}\left(0\right){|}^{2}>$$

where $r_{i}\left(0\right)$ is the original position for atom *i* and $r_{i}\left(t\right)$ represents the position for atom *i* at time *t*, *N* is the number of atoms in the system.

**Figure 11:**
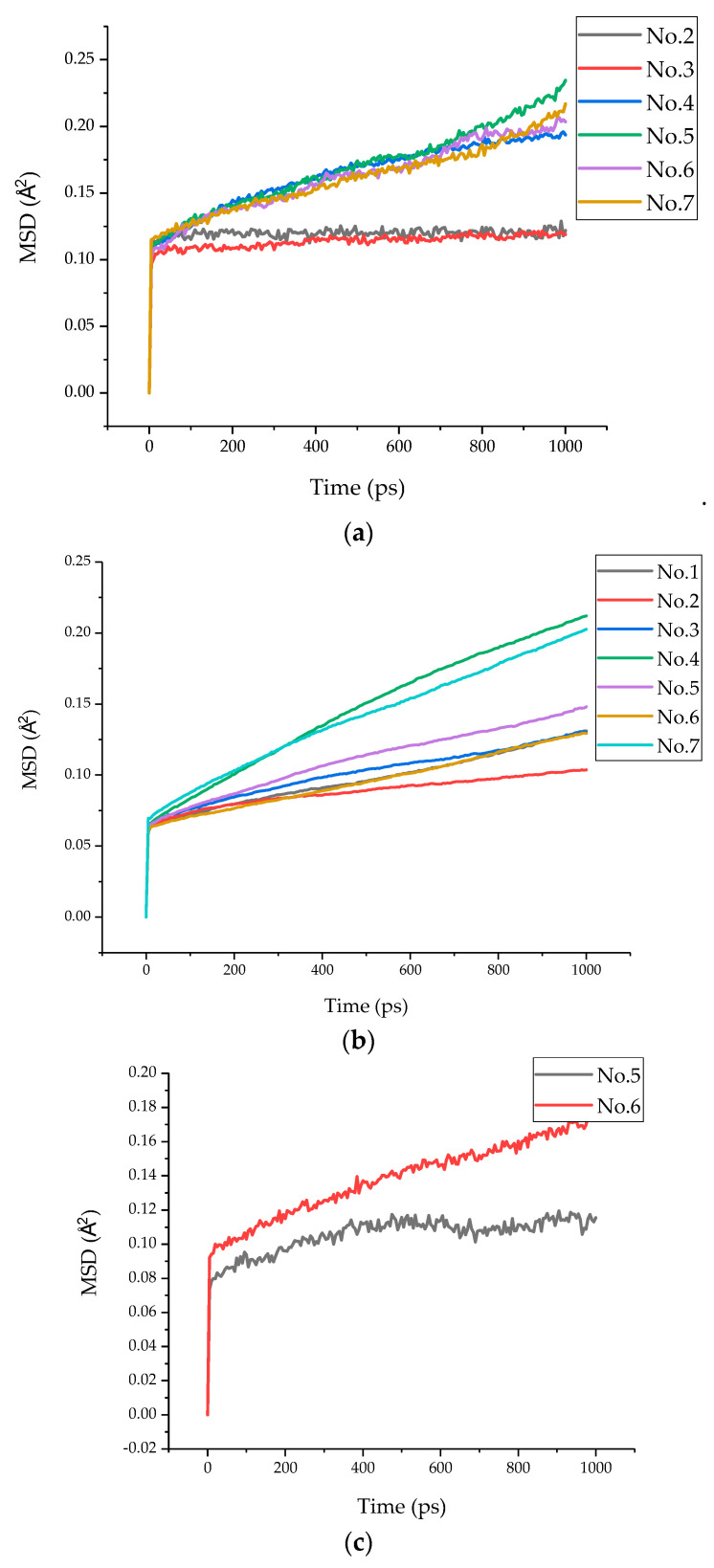
MSD of O, Si, and N atoms. (**a**) MSD of O atoms. (**b**) MSD of Si atoms. (**c**) MSD of N atoms.

In the polymer composite, the MSD for the oxygen atoms in polymers and the O_w_ atoms in water are often used to indicate the faster movement of interlayer species, including the rotation and vibration of the polymer chains and their branch structures [16]. The MSD of oxygen atoms in each model epoxy resin chain are presented in Figure 11a. It showed that the displacements of the six models were 0.12 Å, 0.12 Å, 0.19 Å, 0.23 Å, 0.20 Å, and 0.22 Å, respectively. It can be clearly seen from the MSD curves of models 2, 3, and 4 that when the chain length was 0 or 1 (models 2 and 3), the displacement was still the same; when the chain length increased to 2 (model 4), a noticeable increment on the MSD curve could be observed, which might be attributed to the increasing chain length enhancing the atomic interaction between the epoxy resin and the tobermorite. Meanwhile, it was found that the MSD also increased when the epoxy resin was cured and the epoxy resin chain was parallel to the *Z axis* (models 5, 6, and 7). This may be because the O atoms in epoxy received more attraction from tobermorite than in model 2 and model 3. The deduction can be further proven with the increased binding energy between layers, which will be discussed in Section 3.3.

Similarly, the MSD of Si atoms in tobermorite is also shown in Figure 11b. It can be seen from the figure that the displacements of the seven models were 0.13 Å, 0.10 Å, 0.13 Å, 0.21 Å, 0.15 Å, 0.13 Å, and 0.20 Å, respectively. Compared with Figure 11a,b, it is obvious that the MSD of the Si atoms was smaller than that of the O atoms, which indicates that tobermorite showed a more stable skeleton function.

Since N-atoms only existed in the epoxy with a curing agent, the MSD of N atoms in the epoxy resin chain in models 5 and 6 are presented in Figure 11c. It is obvious that the MSD of N atoms in model 6 (0.18 Å) was greater than that in model 5 (0.12 Å), which indicates that the curing agent, TEPA, was more affected by the interaction of tobermorite than that by MC120D.

### 3.3. Binding Energy

The different configurations and dynamic behaviour of the composites could be attributed to the change in interaction between the polymers and the substances. Therefore, in this study, the binding energy, which is the opposite of the interaction energy between the tobermorite and the epoxy, was calculated by following Equation (3) [45]:

(3) $$E_{b}=-E_{I}=E_{total}-\left(E_{\mathrm{Tobermorite}}+E_{epoxy}\right)$$

where $E_{b}$ is the binding energy between the tobermorite and the epoxy, $E_{I}$ is the interaction energy, $E_{total}$ is the total energy of the whole model, and $E_{\mathrm{Tobermorite}}$ and $E_{epoxy}$ are the energy of the tobermorite and epoxy, respectively.

The interfacial binding energy between the epoxy resin and the tobermorite on both sides is presented in Table 3. It can be seen that the interfacial binding energy of models 2 and 3 was close, while the interfacial binding energy of model 4 was larger than the two models, which is consistent with the previous investigations on MSD. Thus, it can be concluded that increasing the chain length can strengthen the interaction strength between epoxy resin and tobermorite. Moreover, in models 5, 6, and 7, the type of curing agent and the direction of the chain increased the interfacial binding energy, with significant effect from the curing agent. As presented in Table 3, after being cured with MC120D and TEPA, the interfacial binding energy increased by 21.33 kcal/mol (Model 5) and 31.03 kcal/mol (Model 6), respectively, while it only increased by 8.43 kcal/mol (Model 7) after changing direction. Therefore, it can be safely deducted that the use of a curing agent can strengthen the interfacial binding energy between epoxy resin and tobermorite, with a higher binding energy from TEPA, which is consistent with Zhang’s experimental results [12].

### 3.4. Radial Distribution Function

**Figure 12:**
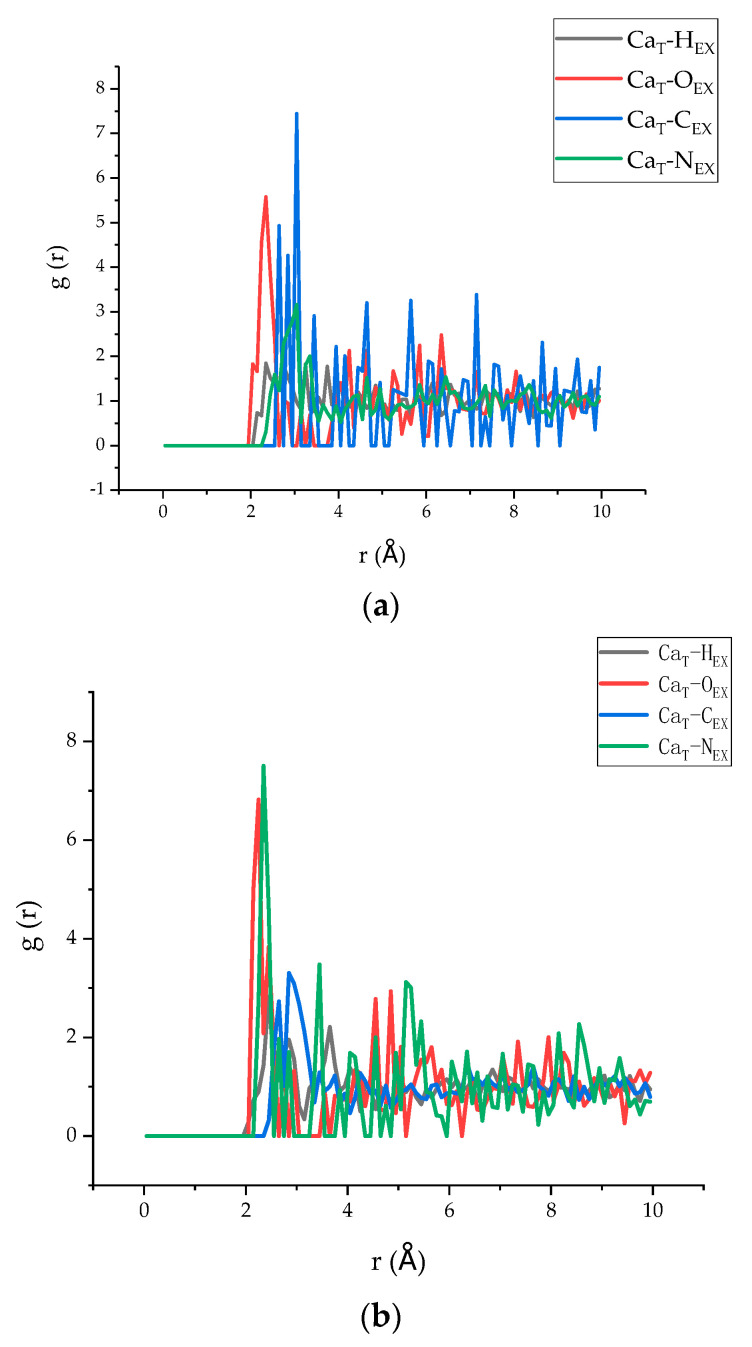
The RDF of models 5 and 6. (**a**) The RDF of model 5. (**b**) The RDF of model 6.

The radial distribution function (RDF), which deals with spatial atomic correlations, can provide abundant structural information for the tobermorite/polymer composites [46]. Therefore, in addition to the analysis in Section 3.3, the RDF of model 5 and model 6 was investigated to analyse the spatial correlation of the different atoms between the two layers. According to the literature, in which Ca atoms are commonly selected to indicate the connection between polymers and C–S–H [47], the RDF of Ca atoms of tobermorite in the models and other atoms in the epoxy resin is shown in Figure 12a,b. Since it is widely accepted that the position of the first peak is related to the connection between the two atoms [48,49], i the lower value of r indicates a stronger connection. It can be seen from the figure that the first peak of Ca_T_–H_EX_, Ca_T_–O_EX_, Ca_T_–C_EX_, and Ca_T_–N_EX_ of model 5 appeared at 2.35 Å, 2.35 Å, 2.65 Å, and 3.05 Å, respectively, while model 6 appeared at 2.45 Å, 2.25 Å, 2.65 Å, and 2.35 Å, respectively. Obviously, Ca–O appears earlier than others, indicating that there is a good spatial correlation between Ca and O. Therefore, the Ca–O bond played a major role in the interaction between the tobermorite and the epoxy resin layers. Moreover, by comparing the two figures, it can be clearly seen that the value of r of the Ca and O atoms of model 6 is smaller than that of model 5, which may suggest that TEPA offered a better interaction between O in the epoxy and Ca in the tobermorite. Furthermore, it can be clearly seen that the appearance of the first peaks of N and Ca in model 6 was earlier than that in model 5, which suggested that N in the TEPA played a better role in connecting with Ca. Therefore, the epoxy resin using TEPA acting as the curing agent had better interfacial binding energy with tobermorite compared with MC120D.

**Figure 13:**
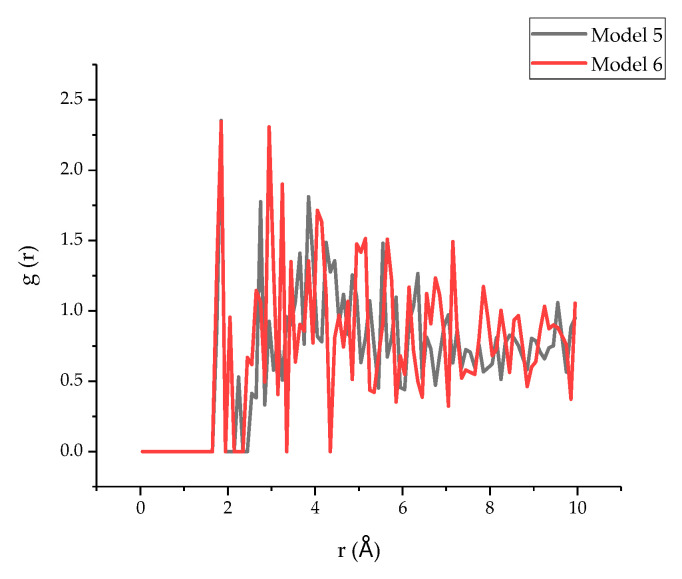
The RDF of H in T and O in epoxy resin.

In addition, because the O and H atoms between the interface may form hydrogen bonds and increase the interfacial binding energy [50], the RDF of H in the tobermorite and O in the epoxy resin in model 5 and model 6 was simulated, and the results are shown in Figure 13. Obviously, the first peak of both models appeared at around 1.75 Å, indicating that the hydrogen bond existed, since, according to the literature, an RDF value between O and H atoms of less than 2.45 Å may indicate the existence of a hydrogen bond [51]. Therefore, based on the current results, it can be deducted that the interfacial binding energy not only consists of the interaction between Ca and other atoms in the epoxy resin, but also of the hydrogen bonding between O atoms and H atoms. Similarly, the positions of first peak of model 5 and model 6 are the same, and the peak height of model 6 (1.31) is greater than that of model 5 (0.88), which further proves a better connection of epoxy with TEPA due to the hydration bond. By calculating the area of the first peak of the RDF, it can be observed that compared with model 5 (0.32), a larger value was obtained in model 6 (0.37), which therefore proved that the hydrogen bond of model 6 was stronger than that of model 5.

## 4. Conclusions

In this work, seven composite models were established to investigate the interfacial binding energy between C–S–H and epoxy resin via molecular dynamics simulation. Based on the simulation, the following conclusions can be summarized:

(1) After the molecular dynamic simulation, the layers of the three-layer structure of the tobermorite and epoxy resin in the model permeate each other due to the interaction between atoms, and the cracks between the models were reduced.
(2) The MSD results showed that there was a larger displacement of O in the cured epoxy, which indicated the strong mutual attraction between epoxy resin and tobermorite. Meantime, the N atom in the TEPA showed a larger displacement than that in MC120D, revealing that TEPA can offer better binding behaviour between the epoxy resin and the cementitious matrix.
(3) Based on the MD simulation, the incorporation of a curing agent significantly increased the binding energy, with a higher value of TEPA (50.53 kcal/mol) suggesting that the system of epoxy with a TEPA curing agent may provide a better repairing performance.
(4) According to RDF analysis, the earliest appearance of the first peak of Ca in tobermorite and the O in epoxy was observed, indicating the strongest interaction between O atoms and Ca atoms. Additionally, the RDF of Ca in tobermorite and N in cured epoxy further confirmed that the epoxy cured by TEPA can offer a better binding effect on repairing cementitious materials. In addition, O and H atoms between the interfaces will form hydrogen bonds, thus increasing the interfacial binding energy.
(5) The results revealed that the chain length and chain direction showed limited influence on the binding energy between the epoxy and the tobermorite, while the type of curing agent significantly boosted the binding energy. Based on the simulation, it exhibited a better ability to repair cracks in cementitious material.

Although the binding effect between epoxy resin and a cementitious matrix has been simulated, which may guide the selection of a curing agent to offer better repairing performance, an experimental investigation should be conducted to verify the simulation.

## Figures and Tables

**Table 1:** Parameters of seven simulation models.

Model	*n*	Curing Agent	Direction
No. 1	N/A	N/A	N/A
No. 2	0	N/A	X
No. 3	1	N/A	X
No. 4	2	N/A	X
No. 5	0	MC120D	X
No. 6	0	TEPA	X
No. 7	1	N/A	Z

Note: model 1 has no epoxy resin inserted. *n* is the degree of polymerization of the epoxy resin. Additionally, the direction refers to the direction of the epoxy chain in the model.

**Table 2:** Cell lengths in the Z direction.

Model	Before MD Simulation (Å)	After MD Simulation (Å)	Reduction (Å)
No. 1	68.72	61.33	7.39
No. 2	65.2	59.48	5.72
No. 3	67.93	61.41	6.52
No. 4	67.89	61.44	6.45
No. 5	68.1	61.55	6.55
No. 6	64.97	59.49	5.48

**Table 3:** Binding energy.

Model	$E_{b}\;(\mathrm{kcal}/\mathrm{mol})$
No. 1	N/A
No. 2	19.5
No. 3	18.36
No. 4	23.3
No. 5	40.83
No. 6	50.53
No. 7	27.93

## Data Availability

Data available on request.
